# The Application of Hypnosis in Sports

**DOI:** 10.3389/fpsyg.2021.771162

**Published:** 2022-01-24

**Authors:** Zhe Li, Su-Xia Li

**Affiliations:** ^1^Chinese Health and Humanity, Department of History, University College London, London, United Kingdom; ^2^National Institute on Drug Dependence, Beijing Key Laboratory of Drug Dependence, Peking University, Beijing, China

**Keywords:** hypnosis, athletes, anxiety, relaxation, sports

## Abstract

As an ancient science, hypnosis has been used by humans since the primitive societies during theocratic times. By the 20th century, scientists and psychologists had re-recognized and studied hypnosis and explored its applications in fields such as medicine, education, and military uses. A local form of traditional Chinese “hypnosis” appeared in *Huangdi Neijing*, but it has not received enough attention from Chinese people; China’s modern hypnosis development is later than that of American and European countries. Therefore, people’s understanding and applications of hypnosis remain inadequate. With the development of China’s economy and state power in the last decades, Chinese people were beginning to attach importance to the investment and development of the sports industry and realized that the psychological quality of the athletes is often a decisive factor in the success or failure of the competition. Meanwhile, hypnosis is an effective psychological training method to be used in daily training and competitions. In light of this, this essay firstly reviews the history of hypnosis before carrying out the methods of a literature review and a logical analysis to explore the applications of hypnosis in sports. Thus, evidence is provided in favor of the use of hypnosis in the sports industry.

## A Brief History of Hypnosis

Hypnosis is generally believed to have existed since ancient societies (Hammond, 2013). In China, the earliest recorded text on hypnosis was in the *Huangdi Neijing [written during the Warring States period (475-221 BC) and the Han dynasty (206 BCE–220 CE)]*. It mentioned a healing method called “Zhu You,” which appears to have many elements in common with hypnosis from a modern perspective (Yao, 2010). “Zhu You” was a sort of witchcraft. It used spells to cure diseases. Apart from China, the “Sleep Temples” in ancient Greek and Roma centuries were used to induce a sleep-like state (Muthu, 1930), which can be regarded as the earliest hypnosis-like state. Sleep temples could heal a variety of ailments. Many of them were psychological in nature. The treatment involved chanting, meditation, fasting, placing the patients into a trance-like or hypnotic state, and analyzing their dreams, etc.

Modern hypnosis could be traced back to Franz Mesmer’s (1734–1815) animal magnetism theory in the 1770s. He believed that all things in nature shared a common magnetic force, and disease could be cured through a magnetic field (Hammond, 2013). His mesmerism was regarded as mysterious witchcraft and was not adopted by the scientific community. The mesmerism in that era performed under the exaggerated background of various spells and astrological wall decorations, with mesmerists using various strange props to inspire the souls of sleepwalkers to come and communicate with them (Darnton, 1968). Later, James Braid (1795–1860) ended the old witchcraft era of hypnotism mystification. He believed that hypnosis was not produced from magnetism. It was a kind of sleep-like state and defined as “a peculiar condition of the nervous system, induced by a fixed and abstracted attention of the mental and visual eye, on one object, not of an exciting nature” (Braid, 1843). At the same time, he coined the term “hypnosis” (to sleep) from the ancient Greek term “Hypnos” (the god of sleep in ancient Greek mythology). He also emphasized a theoretical conception of hypnosis as a state of increased suggestibility and a form of sleep (Hammond, 2013). With the in-depth study of hypnosis by scientists, there were two opposing schools in hypnosis academia in the 19th century. They were the Paris School represented by Jean-Martin Charcot (1825–1893) and the Nancy School represented by Hippolyte Marie Bernheim (1840–1919) and Ambroise-Auguste Liébeault (1823–1904). The Nancy School advocated the “suggestion theory,” which believed that the role of suggestion was very important in the process of hypnosis. The phenomenon produced by hypnosis was a reaction caused by the hypnotized person accepting the hypnotist’s suggestion and that the person could be put into a hypnotic state by purely verbal suggestion (William and Edmonston, 1986). The Paris School believed that hypnosis was a form of hysteria, and those who could be hypnotized were psychotic (Sheehan and Perry, 1976). Although there were many different understandings of hypnosis between these two schools, they contributed to the development of hypnosis. Subsequently, scholars such as Sigmund Freud (1856–1939) and Ivan Petrovich Pavlov (1849–1936) were also involved in hypnosis research, making hypnosis a valuable applied science.

## Hypnosis Is a Sort of Scientific Psychotherapy

Hypnosis is a technique of communication using psychological suggestions. Under hypnosis, the hypnotized person is more open to suggestions. The result of hypnosis is that they gain greater control over their own minds and their own actions. In the process of hypnosis, the hypnotist will repeatedly use the auditory, visual and tactile stimuli to induce the hypnotized person into a trance state. In the meantime, the hypnotized person will also be asked to relax, imagine and concentrate on the hypnotist’s suggestions. After the hypnotized person enters the hypnotic state, the hypnotist will implement various treatments to address the patient’s psychological disorders. Nowadays, hypnosis has been used in numerous industries such as medical/psychotherapeutic uses, sports training, military uses, self-improvement, employee training, and police confession. Moreover, studies have shown that the hypnotic effect is closely related to the hypnotized person’s attitude and belief in the hypnotic process (Franquelo et al., 2020, 2021a,b). More and more evidence show that hypnosis is a scientific psychological therapy technique.

## Athletes’ Psychological Stress

Psychological training for athletes is an essential part of sports training because, in any competitive event, there is always the possibility of misplaying and losing. Many of the best athletes have worked hard for decades to perform at the top-level competition but may not always win the championship. At the same time, athletes’ misplay is a sort of publicity trick and a conversation topic among the ground. Therefore, the more higher up into the top elite classes athletes are, the more they are under tremendous psychological pressure. A study from Australian academics mentioned that athletes’ emergence of mental illness is usually at the peak of their competitive careers (Allen and Hopkins, 2015). Moreover, they experience unique stresses, including sport-related stress (Noblet and Gifford, 2002), injuries (Smith, 1996; Appaneal et al., 2009), life away from home (Bruner et al., 2008), and burnout (Gustafsson et al., 2011). For these reasons, it is important to pay attention to athletes’ psychological problems. Hypnosis, used as an adjuvant technique, can help to prevent the mental illness of athletes, but also can improve their training results and competition performance. Sports psychologists may apply hypnosis to athletes’ psychological training because hypnosis may help athletes attain relaxation during practice and competition and help athletics control anxiety and manage stress (Paccagnella, 2004). Meanwhile, hypnosis is a simple and easy way to adjust the physical and mental conditioning without special requirements in terms of venues or equipment. A previous review indicated that hypnosis was effective for improving performance across multiple sports, with the strongest results found for basketball, golf, soccer and badminton (Milling and Randazzo, 2016). Therefore, hypnosis is widely used in sports and competitions.

## The Applications of Hypnosis in Sports

### Hypnosis Can Explore the Potentials of Athletes

Hypnosis appears to restructure cognitive processes that are essential for athletic performance, including self-confidence, attention, and memory. The enhanced positive self-affirmations and rational-emotive strategies are commonly reached through the use of hypnosis (Taylor et al., 1993). Athletes may take advantage of this “believed-in” state to enhance and tap into the potentials of their physical conditions. The result can be achieved by the suggestions that hypnotists convey to them, such as “you can perform well today” or “you can push your limits”. These positive suggestions can help them rebuild their self-confidence, allowing them to concentrate more on their task to reach their goals more easily.

### Hypnosis Can Relieve the Anxiety of Athletes

It is common for athletes to experience nervousness and stress before a significant event. These negative moods and mental strain cannot only affect their daily training and life, but may also affect their performance in competition. Research (Ji, 1992) has shown that stress can affect both physiological and psychological functions. The former includes increased respiratory rate, heart rate, and increased blood pressure; the latter includes blurred perceptions, slowed reactions, and thinking and distracted attention. These negative factors can influence athletes’ performance during competitions. Hypnosis can be used to reduce athletes’ different levels of tension and stress before and during the competition. The hypnotist can give the hypnotized athlete suggestions such as “you may be noticing that you are feeling quieter and quieter and nicer and nicer, and your breathing is getting smoother and smoother,” “you can win the competition.” These suggestions can have a positive effect on athletes both psychologically and physically (Ji, 1992).

### Hypnosis Can Reduce the Fears of Athletes

In gymnastics, fears are an obstacle to athletes’ training and competitions. At the same time, the fears of an individual athlete may develop into a group effect that makes it challenging to continue group training. What is more, fears may lead to athletes’ thinking disorder, inability to concentrate, related muscle tension, increased heart rate and breathing, weakness of the limbs and even collapse (Xu and Cao, 2013). These phenomena can cause distortions in the athletes’ movements and cause boredom to athletes with gymnastics and, in severe cases, accidents and injuries. Fortunately, Reid’s study has shown that hypnosis can relieve fear, stress, anxiety and can be used to help in coping with panic disorder (Reid, 2017). This is because hypnosis makes athletes relaxed and hypnotists will use calming words to encourage them. For instance, “you are safe although your discomfort,” “you can control your mind.” The hypnotist will also suggest ways for athletes to cope with their fears, such as “taking deep breaths when you are afraid of training makes you feel calmer.” In this way, hypnosis provides athletes an ideal chance to learn how to remain relaxed while facing these fears and performing at the competitions.

### Hypnosis Can Help Athletes Learn and Improve Their Technique

The enhancement of individual techniques and tactics of athletes not only relies on the improvement of muscle strength and physical qualities, but also depends on athletes’ mentality. Therefore, any physical activity is a brain-body combination. Sports psychologists place particular emphasis on imagining skills training for athletes as it helps them build on their strengths, eliminate their weaknesses, develop their sports skills, stay confident, focused, and motivated (Ji, 1992). Athletes are often encouraged to imagine the critical event in their training and identify with the performance as much as possible. They could note everything influencing them, their thoughts, their physical experiences, their competitors, and other significant things. This process can help athletes become aware of aspects of the performance experience that are unnoticed, and this can lead to the discovery of key information. Moreover, it is evidenced that hypnosis can enhance the imagining skills’ quality on self-efficacy, skill acquisition, and athletic performance (Taylor et al., 1993). Due to the relaxed state that athletes are when they are hypnotized, the results of imagining skills training are better than that of training in a waking state, which can help athletes gain and improve their individual technique more efficiently. Even a 10-min hypnosis intervention can improve throwing accuracy in a tennis ball test and the retention of the effect can last for a 1-week (Jalene and Wulf, 2014).

Be it from multiple or one hypnotic intervention, hypnosis has been found to have positive effects on ball games, including basketball, golf, soccer, cricket, and badminton (Pates and Maynard, 2000; Pates et al., 2001a,b, 2002; Pates and Palmi, 2002; Barker and Jones, 2006, 2008; Barker et al., 2010; Pates, 2013), and other sports, including archery, weightlifting, shooting sports (Lee Howard and Reardon, 1986; Robazza and Bortoli, 1995; Mattle et al., 2020), in either a control study or a single case study. There have also been conflicting reports of studies that had not found a positive effect of hypnosis on athletic performance, such as in tennis (Greer and Engs, 1986), fencing (Wojcikiewicz and Orlick, 1987), and boxing (Heyman, 1987). However, these studies have defects such as small sample size or poor design, which require further research in the future.

### Hypnosis Can Help Athletes Quickly Relieve Fatigue and Restore Energy

The training athletes undergo is continuous and of high intensity, which can lead to frequent muscle soreness and physical exertion. Therefore, athletes usually require adequate sleep, massage, hydrotherapy, and nutritious meals to restore their physical strength and energy. However, during competition, fatigue and physical exertion can be decisive factors in the success or failure of the competition. If an athlete has more energy than his opponent, he may also have a better chance of winning than his opponent. Therefore, it is vital to replenish energy and restore physical strength during the competition quickly. Hypnosis can quickly relieve fatigue and restore physical strength (Niu, 2013); it can be performed before or between training and match. When the athlete enters the hypnotic state, their muscles can be fully relaxed. The suggestion can be “you have entered the state of hypnosis, your muscles are fully relaxed, and the fatigue will be relieved immediately by the massage.” During the process, suggestion can be “fatigue has been relieved and that your energy and strength have now been restored,” “you have had three (or more) hours of rest, the fatigue has all been relieved and you feel energetic and strong again,” “continue to join in the match, your energy and physical strength will be very abundant.” These positive suggestions can help athletes quickly replenish themselves and gain more confidence.

### Hypnosis Can Help With Sleep

It is natural that athletes could be nervous before a competition, especially when facing a competition related to their country’s reputation. They could tend to have difficulty falling asleep due to extreme tension. Insufficient sleep will affect them and hinder them from replenishing their bodies and restoring their strength, which will affect their performance in the next day’s game. There had been several studies on the procedures involving relaxation help sleep (Graham et al., 1975), and scientists found that procedures involving relaxation can play an influential role in reducing the time required for subjects to fall asleep. Hypnosis can help athletes enter a relaxation state. Hypnotists will help athletes with sleep problems by encouraging them to relax and creating them an opportunity to reorient thoughts and emotions (Becker, 2015). Studies have shown that modest sleep can benefits from hypnosis, and the suggestion of “sleep deeper” can help participants prompted increased slow-wave sleep (Feng, 1995). Slow-wave sleep often is referred to as deep sleep. This period of sleep is called slow-wave sleep because the EEG activity produces slow waves with a frequency of less than 1 Hz and a relatively high amplitude. Hypnosis may also reduce symptoms such as anxiety and depression as well as treat pain, and these factors are strongly related to sleep problems or can cause disturbed sleep.

## Limitations and Prospects

At present, there are several defects in hypnosis application in sports. Firstly, in most studies, the sample size was small. Some had fewer than 10 subjects. Secondly, the design of the research scheme is not rigorous enough. For example, some studies were just pre - and post-comparison studies without different types of hypnosis for comparison. The evaluators of hypnosis effect were not blind to the research scheme. Results could easily be influenced by subjective factors when evaluated. Thirdly, no randomized placebo-controlled studies have been found. Fourth, there are very few studies on the effect of hypnosis on performance in competitions. In the future, studies on the application of hypnosis in sports can be appropriately conducted with large sample sizes, setting up different types of hypnosis for comparison, blinding the evaluators of effects of hypnosis, and setting up reasonable and scientific placebo control. In addition, the effect of hypnosis on performance in competitions should also be further studied.

## Conclusion

There is a lot of evidence that hypnosis may be a helpful tool to address a variety of issues in sports; for example, hypnosis may help athletes improve their psychological quality, increase their self-confidence, relieve their fatigue, restore energy, help them better concentrate on the daily training and competition, and better improve their skills. However, there is little methodologically rigorous empirical research substantiating its value, and there is still a lack of extensive experimental evidence to prove its mechanism. As a result, hypnosis in sports still needs to be studied in more depth and breadth, and empirical verification is necessary and required. But it is undeniable that hypnosis is a simple and easy way to adjust physical and mental conditioning without special requirements in terms of venues or equipment.

## Author Contributions

ZL contributed to the literature search, interpretation, conceptualization, and drafted the manuscript. S-XL contributed to conceptualization, interpretation, and final approval of the manuscript. Both authors contributed to the article and approved the submitted version.

## Conflict of Interest

The authors declare that the research was conducted in the absence of any commercial or financial relationships that could be construed as a potential conflict of interest.

## Publisher’s Note

All claims expressed in this article are solely those of the authors and do not necessarily represent those of their affiliated organizations, or those of the publisher, the editors and the reviewers. Any product that may be evaluated in this article, or claim that may be made by its manufacturer, is not guaranteed or endorsed by the publisher.
